# Exosomes secreted by human cells transport largely mRNA fragments that are enriched in the 3′-untranslated regions

**DOI:** 10.1186/1745-6150-8-12

**Published:** 2013-06-07

**Authors:** Arsen O Batagov, Igor V Kurochkin

**Affiliations:** 1 Department of Genome and Gene Expression Data Analysis, Bioinformatics Institute, 30 Biopolis str #07-01, Singapore 138671, Singapore

**Keywords:** Exosomes, Secreted RNA, 3′-UTR, Microarray analysis

## Abstract

**Abstract:**

Small secreted membrane vesicles called exosomes have recently attracted a great interest after the discovery that they transfer mRNA that can be translated into protein in recipient cells. Surprisingly, we found that for the majority of exosomal mRNAs only a fraction of their corresponding probes is detectable on the expression microarrays. Exosomal mRNA fragmentation is characterized with a specific structural pattern. The closer to the 3′-end of the transcript the fragments are localized, the larger fraction among the secreted RNAs they constitute. Since the 3′-ends of transcripts contain elements conferring subcellular localization of mRNA and are rich in miRNA-binding sites, exosomal RNA may act as competing RNA to regulate stability, localization and translation activity of mRNAs in recipient cells.

**Reviewers:**

This article was reviewed by Neil Smalheiser and Sandor Pongor.

## Findings

Exosomes are small nano-sized (50–150 nm) membrane vesicles secreted by most cell types including hematopoietic, neuronal, fibroblastic and various tumor cells [1]. Discovered 25 years ago [2], they were thought to be involved in just discarding unwanted cellular debris. Later research, however, uncovered their role as essential cell-to-cell communication vehicles that function via addressed delivery of specific sets of proteins and bioactive lipids [1]. These vesicles have recently attracted a great interest after the discovery that they contain mRNA [3,4], microRNA [3-5] and DNA [6]. Interestingly, the RNA patterns of exosomes were found to be substantially different from their host cells. Many of the mRNAs and miRNAs were highly enriched or even exclusively present in exosomes suggesting an existence of a dedicated mechanism for selective targeting of the RNAs into these vesicles [3,4]. We recently identified several linear motifs highly enriched in secreted RNAs and proposed that their combination within a given RNA defines a zipcode recognized by trans-acting factors targeting RNAs to exosomes [7]. Exosomes are present in various body fluids and expression profiling of their RNA in blood plasma, for example, could differentiate between healthy controls and patients with certain types of cancer [8] demonstrating their potential value as biomarkers. Exosomal mRNAs could be transferred to other cells in culture dish experiments [3,4]. Moreover, in one report [3] host cell-derived exosomal mRNA was functional as it could be translated into proteins in target cells. The ability of exosomes to deliver mRNA to cells at a distance suggests their potential role in altering the recipient cell protein production [3]. Intact mammalian mRNAs vary in length from 400 nt to 12,000 nt with the average size of transcripts 2,100 nt [9]. However, the majority of investigated normal and cancer cells secrete exosomal RNAs with a size distributed between 25 and 700 nt. For example, RNA of a small size (<700 nt) was present in human plasma [10], saliva and breast milk exosomes [10,11]. Human mesenchymal stem cells [12] and human tracheobronchial epithelial cells [13] were found to secrete even smaller RNA species (< 500 nt in length).

One possible explanation for this observation could be that exosomes are enriched in mRNAs encoding very short proteins. However, Frith with colleagues [14] analyzed RNA sizes for different ranges of proteins and found that the center of the RNA length distribution is almost same (around 2,100 nt) for large (>300 amino acids) and short (<100 amino acids) proteins. Thus, the size distribution of exosomal RNA suggests that the most of the RNA molecules present in these vesicles consist of species intermediary in a length between mature miRNAs (22 nt), pre-miRNAs (70 nt) and full-length mRNAs. The simplest explanation for this size distribution would be that exosomes are enriched in truncated mRNAs. Recent studies established that RNA transcripts may undergo a widespread post-transcriptional cleavage producing a range of smaller coding and noncoding RNAs [15]. Post-transcriptional RNA cleavage appears to be a tightly controlled process as it is highly tissue-specific and developmentally regulated [15].

Next generation sequencing-based method RNA-Seq allows accurate determination of transcript boundaries and thus could be used to verify the hypothesis that exosomes carry RNA fragments. However, in case of exosome microvesicles, this approach was applied only for the analysis of small RNAs [16,17]. Interestingly, these studies uncovered that exosomes contain a large number of transfer-, vault- and Y-RNA fragments [16,17].

**Figure 1:**
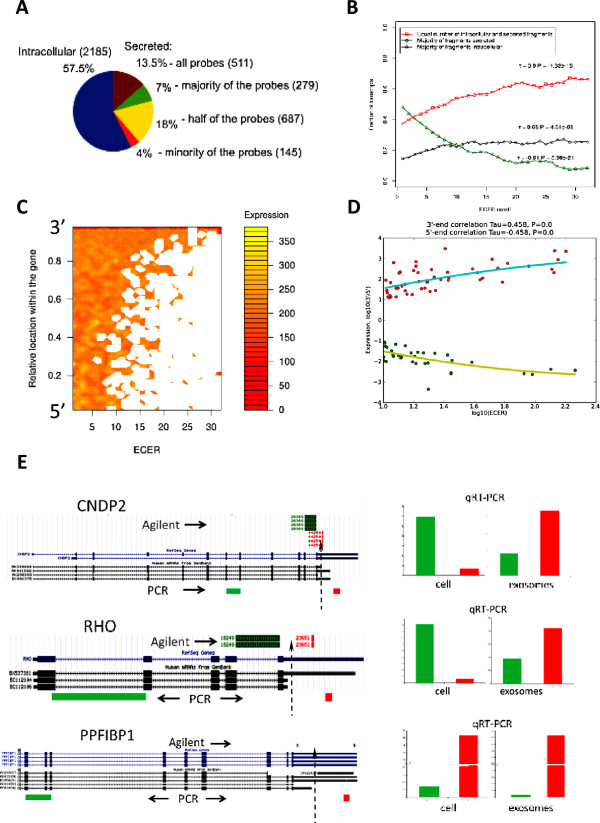
**Detection of mRNA fragments in exosomes secreted by human cells. (A)** Distribution of the transcripts by exosomal secretion of their fragments. Secreted transcripts were identified with ECER ≥ 3. Transcripts with all secreted probes were considered as secreted unfragmented. Fragmented transcripts were classified into three classes with i) majority, ii) minority and iii) exactly half of the probes secreted. **(B)** Cumulative fraction of fragmented transcripts in the total RNA measured in exosomes versus secretion magnitude (ECER cutoff). **(C)** Distribution of individual probe expression in exosomes by the magnitude of their secretion (ECER) and their location. The probes measure exosomal expression of specific fragments of the transcripts. Expression level is depicted with color ranging from red (low expression) to yellow (high expression). Relative location of the probes within their transcripts is represented by a number ranging from 0 (5’-end) to 1 (3’-end) with precision step 0.02 of the total relative length (1.0) of a transcript. **(D)** Dependence of probe localization relative to the 3’- and the 5’-ends of each individual transcript on the magnitude of its secretion (ECER). Only strongly secreted transcripts (ECER ≥ 10) are shown. Each dot represents a representative probe pair for an individual transcript (see details in Methods section). **(E)** Genomic view of CNDP2, RHO, and PPFIBP1 genes, along with the qPCR results for the SF295 intracellular and exosomal samples. The position of Agilent probes and the amplicons generated by PCR are shown in green and red. Expression was quantified by ΔCT between the genes of interest and that of a firefly luciferase cDNA spike-in control (See Additional file 3: Table S2 and Additional file 5). The potential post-transcriptional cleavage sites are designated by long dashed arrows.

To detect possible presence of mRNA fragments in exosomes we utilized a microarray dataset from the published study [4] that analyzed mRNA content of exosomes released by cultured glioblastoma primary cells. The microarray analysis of mRNA was performed using the Agilent whole genome microarray whose 60-mer oligonucleotide probes are designed in a way that allows interrogation of expression levels of various RNA regions. We analyzed gene expression in cells and their secreted exosomes probe-wise. RefSeq transcripts were classified by the presence of signals from their microarray probes in exosomes and within the cells into four classes: i) 511 transcripts for which all the probes targeting each transcript were secreted from cells via exosomes, ii) 687 transcripts for which exactly half of the probes were secreted and half retained in the cell (including 656, 27 and 4 with 1, 2 and 3 secreted/retained probes, respectively), iii) 279 transcripts for which more than a half of the probes was secreted, iv) 145 transcripts for which less than a half of the probes was secreted (Figure 1A).

Classes ii-iv, representing putative transcripts secreted in fragments, constituted 68.5% of all secreted transcripts. Moreover, we observed that with increase of the enrichment in exosomes the fraction of partially secreted transcripts of both classes increased (Figure 1B). The fraction of transcripts with exactly half of the probes secreted demonstrated almost two-fold increase (from 37% to 66% ) and positively correlated with the secretion efficiency (τ = 0.90). In contrast, the fraction of secreted intact transcripts decreased more than seven-fold (from 36% to 5% ) and negatively correlated with the secretion (τ = −0.91). These results suggest that transcript fragmentation and secretion are inter-related.

We performed the analysis of gene ontologies (GOs) associated with each class of secreted transcripts (See Additional file 1: Table S1). The protein products of the fragmented mRNAs were found to be significantly enriched in enzyme modulation (*P* = 0.0013) and in proteins participating in extracellular transport (*P* = 0.028). On the other hand, the proteins encoded by the full-length secreted mRNAs are specialized at cell surface receptor linked signal transduction (*P* = 2.04∙10^-4^), cell communication (*P* = 8.06∙10^-4^) and system development (*P* = 0.029). Interestingly, the products of 17 of these transcripts are localized in the extracellular matrix (*P* = 0.0046). Thus it can be concluded that secreted transcript fragments might have specific functions.

We observed that secreted RNAs are characterized with a specific segmentation pattern. The larger was the enrichment of transcripts in exosomes (ECER), the stronger was the tendency of the probes to be localized in the 3′-end of the transcripts (Figure 1C). The proximity of the probes to their transcript’s 3′-end strongly positively correlated with their secretion (τ = 0.458, *P* < 0.001, Figure 1D). When the transcripts with most fragments secreted were studied separately, a strong positive correlation with secretion was observed only for the probes located at their untranslated regions (UTRs) (τ = 0.73, *P* = 6.86∙10^-9^) (See Additional file 2: Figure S1A). For probes in the translated regions, such correlation was weak (τ = 0.27, *P* = 0.03) (See Additional file 2: Figure S1B). Even larger difference was observed in the fraction of strongly secreted transcripts (*ECER* ≥ 10), with the probe localization in the UTRs correlating with secretion positively (τ = 0.51,P = 1.04⋅10^−3^) (See Additional file 2: Figure S1C) and localization in the translated regions correlating negatively (τ = −0.3, *P* = 0.047) (See Additional file 2: Figure S1D).

To validate the accuracy of the results obtained with the microarrays, we examined the presence of various transcript parts in exosomes using quantitative real-time PCR (qPCR). RNA was isolated from exosomes secreted by human glioblastoma cells SF295. Three exemplary mRNA targets were selected for qPCR analysis, for which we observed unequal distribution of probe intensities on the microarray between cellular and exosomal RNA - CNDP2, RHO, PPFIBP1 mRNAs (See Additional file 3: Table S2). The analysis revealed that the ratio of the amount of qPCR products specific for the 3′-ends to that for the 5′-ends was significantly higher in exosomal fraction suggesting predominat secretion of the 3′-end derived fragments of these thranscripts (Figure 1E, Table S2).

The fact that exosomes carry the 3′-UTRs of mRNAs may have important implications for the regulation of gene expression and protein translation in recipient cells. The 3′-UTRs of mRNAs are rich in regulatory sequences. They serve as binding sites for numerous RNA-binding proteins that modulate stability and translational efficiency of mRNAs. They also contain miRNA target sites that guide the RNA-induced silencing complex (RISC) to microRNA response elements on target transcripts resulting in their degradation or translational reppression. A single 3′-UTR contains many miRNA binding sites. We can imagine that the 3′-UTR derived mRNA fragments carried by exosomes could directly compete for binding of miRNA or specific RNA-binding proteins to the recipient cell mRNA and lead to deregulation in protein production. Lee et al. reported that expression of versican 3′-UTR induces organ adhesion in transgenic mice through binding miR-199a* and freeing mRNAs of versican from being repressed by miR-199a* [18]. The 3′-UTRs could affect not only mRNAs from which they are derived but also mRNAs that share with them miRNA-binding sites. For example, computational analysis indicated that miRNAs that interact with the CD44 3′-UTR also have binding sites in other matrix encoding mRNA 3′-UTRs, including collagen type 1α1 (Col1α1) repressed by miR-328 and fibronectin type 1 (FN1) repressed by miR-512-3p, miR-491 and miR-671 [19]. Transfection of the CD44 3′-UTR led to synergestic up-regulation of CD44, Col1α1, and FN1 proteins and as result enhanced cell motility, invasion and cell adhesion [19]. Pandolfi and colleagues proposed that RNAs ability to compete with each other for miRNAs generates a large-scale trans-regulatory crosstalk across the transcriptome as a whole. They named this RNA network activity “competing endogenous RNA” language [20]. It is tempting to speculate that exosomes may utilize this RNA language as a means of communication between cells to integrate a complex network of information in multicellular organisms. By gaining a more detailed knowledge of the intercellular RNA language it will be possible to make useful predictions on the regulatory roles of RNA species carried by exosomes. It is unclear at present what mechanism might be responsible for generation of the exosomal 3′-UTR containing fragments. Mercer et al. [21] provided an evidence for the existence of a large number of intracellular 3′-UTR-bearing RNA fragments in human and mouse that are expressed separately from the associated protein-coding sequences to which they are normally linked. The post-transcriptional cleavage of mRNAs, rather than new transcription initiation, was proposed to be a major mechanism for the 3′-UTRs production [21]. Regarding the site of mRNA fragmentation, we cannot exclude a possibility that the fragments are generated after secretion by RNases originating from donor cells and incorporated into exosome vesicles. We, however, believe that fragments are produced inside the cells. We noted, for example, that three transcripts selected for RT-PCR validation experiment, CNDP2, RHO, PPFIBP1 are present in various cDNA libraries not only in their full length forms but also as smaller transcript isoforms truncated at 3′-UTR, as well as, fragments derived entirely from 3′-UTR (Figure 1E).

In addition to controlling translation efficiency of mRNAs, the 3′-UTRs are also critical for the subcellular localization of mRNAs [22]. The 3′-UTR fragments transported by exosomes might thus act as decoys to titrate trans-acting proteins recognizing localization elements and thereby affect recipient cell mRNA localization. This might serve as a mechanism of relocating proteins synthesis to different subcellular compartments.

In summary, our results provide evidence that exosomes secreted by human cells transport largely mRNA fragments derived from the 3′-ends of mRNA. This finding suggests the need to reassess the assumption that RNA messages delivered by exosomes are mainly translated into proteins by the recipient cells. Instead, we propose that RNA delivered by exosomes play largely regulatory roles. The secreted mRNA may act as competing RNAs to regulate stability, localization and translational activity of mRNAs in target cells, because 3′-UTRs contain elements that confer subcellular localization of mRNAs and are rich in miRNA-binding sites.

## Methods

Expression data, measured with Agilent human gene expression G4112F microarray, were obtained from [4]. Microarray probes overlapping with RefSeq (v.54) genes, each of which overlaps with at least, two probes, were selected. For every selected probe exosome to cell enrichment ratio (ECER) was calculated as the ratio of the mean expression of the probe in exosomes to its mean expression within the cell, as well as relative location within the coordinates of the probe-containing gene, as described previously [7].

For every selected gene the number of probes enriched in the exosomal and cellular fractions were determined at a given ECER cutoff level. We confirmed absence of correlation between probe expression and its localization for weakly secreted and intracellular transcripts (ECER < 3) (See Additional file 4: Figure S2A) and extracellular (ECER ≥ 3) (See Additional file 4: Figure S2B) transcripts, in order to ensure that our further analysis of the relation between the relative location of the probe and secretion of the respective transcript fragment is not affected by probe design [23]. For each transcript two probes, with the highest and the lowest ECER, were selected and their relative location in respect to the 3′ and the 5′ end of the gene was determined. Expression of the probe localized closer the 3′-end was assessed and its ratio to the expression of the other probe (denoted as 3′/5′ ratio) was calculated (Figure 1D).

Gene ontologies were analyzed using Panther database and statistical model [24] using official gene symbols as primary entries. Bonferroni correction for multiple comparisons was applied to the P-values. Correlations were estimated using Kendall’s τ coefficient.

The experimental procedures - isolation of exosomes, RNA extraction and quantitative real-time PCR – are detailed in Additional file 5.

## Reviewers’ comments

### Reviewer 1: Prof. Neil Smalheiser

**Reviewer’s comment:** This article reports that exosomal RNAs which align to mRNAs show a bias towards 3′-UTR regions. This is interpreted as evidence that 3′-UTR fragments are selectively packaged and secreted within exosomes.

I have two major problems with this: First, they have not ruled out an alternative hypothesis, namely, that exosomes initially contain intact mRNAs but there is partial degradation and preferential stability of 3′ fragments (possibly due to binding by the abundant RNA binding proteins present therein). The methods for collecting and isolating exosomes do not employ RNAse inhibitors, and there are no internal controls to monitor the extent of RNA degradation. I have seen (in other studies) great variation in the ribosomal RNA profiles of isolated exosomes, ranging from intact 28S and 18S RNA to total absence, and I strongly suspect that is due to RNA degradation since (in our own unpublished data) we find abundant partial rRNA reads in exosomal fractions.

**Authors’ response:** We thank the reviewer for critical reading of the manuscript and raising several very helpful comments. Indeed, alternative explanation for the observed preferential stability of 3′ fragments would be the partial degradation of exosomal mRNA with RNA binding proteins protecting 3′fragments. However, RNases present in cell culture conditioned medium are unlikely to contribute to mRNA degradation. Several studies (including our unpublished data) have demonstrated that RNA in exosomes is well protected from the attack by exogenous RNases. In the present study, we utilized a microarray dataset from the study analyzing mRNA in exosomes released by cultured glioblastoma primary cells (Skog et al. Nat Cell Biol 2008, 10:1470–1476). RNase treatment of the glioblastoma exosomes in that study led to a very insignificant (less than 7%) decrease in RNA suggesting that exosomal RNA is inaccessible for the RNase from outside the vesicles. We cannot exclude though a possibility that RNases originating from donor cells could be incorporated inside exosome vesicles. So far, the presence of RNases inside exosome vesicles has not been investigated and this poses a question that remains to be addressed. Regarding the levels of intact 28S and 18S RNA found in various exosome preparations, indeed it is very variable. Interestingly, in line with the reviewer’s observation, analysis of several exosome preparations in our lab revealed that even when 18S and 28S intact rRNA peaks were barely detected (using Bionalyzer), rRNA sequences represented the majority of the reads in the RNA-Seq data suggesting that rRNA is extensively fragmented (unpublished data). It is, however, unclear whether the fragmentation is non-specific and occurs randomly or is purposeful and occurs at specific sites in rRNA. Another example is tRNA. It is represented in exosomes mainly by its fragments and the most abundant tRNA hits in exosomal RNA are all located at the 5′end of mature tRNAs (Nolte-’t Hoen et al. Nucleic Acids Res 2012, 40:9272–9285). Regarding the site of mRNA fragments generation, we believe it is likely to be intracellular. We noted, for example, that all three transcripts selected for RT-PCR validation experiment, CNDP2, RHO, PPFIBP1 are present in various cDNA libraries not only in their full length forms but also as smaller transcript isoforms truncated at 3′-UTR, as well as, fragments derived entirely from 3′-UTR (see Figure 1E). The potential post-transcriptional cleavages have been now indicated by the long punctuated arrows in Figure 1E. The above mentioned comments have been added in the new version: *“The post-transcriptional cleavage of mRNAs rather than new transcription initiation was proposed to be a major mechanism for the 3′UTRs production*[21]*. Regarding the site of mRNA fragmentation, we cannot exclude a possibility that the fragments are generated after secretion by RNases originating from donor cells and incorporated into exosome vesicles. We, however, believe that fragments are produced inside cells. We noted, for example, that three transcripts selected for RT-PCR validation experiment, CNDP2, RHO, PPFIBP1 are present in various cDNA libraries not only in their full length forms but also as smaller transcript isoforms truncated at 3′-UTR, as well as, fragments derived entirely from 3′-UTR (*Figure 1E*).”* Regardless the site and the cause of mRNA fragmentation, the preferentially produced 3′-end fragments, we believe, have the potential to act as competing RNA to regulate stability, translation activity and localization of mRNAs in recipient cells.

**Reviewer’s comment:** The second problem with their data is that they have not defined the size distribution or specific lengths of specific 3′-UTR fragments, which would be suggestive of specific processing (though would not totally rule out the possibility of partial degradation on top of discrete protection by RNA binding proteins). They cite Mercer et al. as providing a precedent for the existence of discrete 3′-UTR RNA fragments, but Mercer et al. did define specific lengths, and this should be done here too.

**Authors’ response:** Indeed, Mercer *et al.* study provided defined specific lengths of mRNA fragments because they are based on the data obtained by sequencing of cDNA libraries. Our analysis has been based on previously published microarray data which have a limitation of interrogating levels of mRNA based on hybridization of small probes (60 oligomer in case of Agilent) to different parts of mRNA. Since the probes were designed to match few specific regions of the target mRNAs, their coverage of the transcripts is essentially incomplete and too sparse to use them as positional markers for direct estimation of the transcripts’ sizes.

Nevertheless, considering the importance of the raised question, we performed an additional analysis using the available data. We attempted to estimate the upper bound of fragment lengths by treating the locations of Agilent probes and gene borders as limits. We observed a large variation in the fragment lengths originating from two sources: the natural variation of UTR lengths (ranging from a few hundred to a few thousand nucleotides) and the design of the probes (the probes are distributed highly unevenly within transcripts and there is no common pattern in probe location among different transcripts). The combination of these two factors resulted in over-representation of transcripts with atypically longer UTRs (more than 1000 nt long) among the ones for which fragment length could be reliably estimated (at least, 3 probes per UTR). At ECER cutoff 2 (moderately secreted fragments), within UTRs up to 1000 nt long the median fragment length 122 nt was observed with IQR = 168.25. At ECER cutoff 3 (strongly secreted fragments), the median and the IQR were 60.5 and 32.5 respectively. Understanding the limitations on the biological interpretation of this result, we decided not to include them in the text of the manuscript.

**Reviewer’s comment:** A minor issue is that having 9 supplemental files are too many and not very crucial to their story.

**Authors’ response:** We combined the former Figures S1, S2, S3 and S4 figures into a single file, Figure S1, and renamed them S1A, S1B, S1C and S1D, respectively. We also combined the former Figures S5 and S6 into a single file, Figure S2, and renamed them S2A and S2B, respectively. As the result, the number of supplemental files is 5 in the new version.

**Reviewer’s comment:** My concerns have not been addressed; the revised ms. is essentially the same as the original version.

**Authors’ response:** We appreciate the reviewer’s time to revise our manuscript. His previous comments were most helpful to improve the manuscript and strengthen the biological insights gained from this study. We understood that Reviewer 1 raised no concerns regarding the main results and conclusions. His concerns touched upon the possible mechanisms of mRNA fragmentation and specific lengths of the fragments. Our study did not focus on the causes of mRNA fragmentation but rather on establishing the fact that the majority of exosomal mRNA is fragmented and possible regulatory roles of 3′UTR-derived mRNA fragments in the recipient cells. Regarding the second concern of determining the specific lengths of the fragments, in our reply we described intrinsic limitations of the used experimental platform (microarrays) for such sort of analysis. Nevertheless, we estimated the upper bound of fragment lengths by treating the locations of Agilent probes and gene borders as limits. The numbers were provided in our reply to this comment, but the data were not included in the main text, again, because the platform is not suitable.

We have accepted nearly all the reviewer’s suggestions and have revised the paper accordingly. Specifically, we provided an evidence that exosomal RNA is inaccessible for the RNases from outside the vesicles. Moreover, we provided several potential scenarios of mRNA fragmentation that were incorporated into the revised version of the manuscript. We also modified Figure 1E to indicate the potential intracellular cleavage sites in our validated transcripts.

Reviewer 2: Prof. Sandor Pongor

**Reviewer’s comment:** Exosomes and other microvesicles released by prokaryotic and eukaryotic cells have been known or suspected already early in the 20th century. Their functional properties have only recently reached the focus of rigorous investigation as it was discovered that they can also transfer RNA and one seminal paper showed by microarray studies that this RNA can be translated into proteins by the recipient cell. In this analysis, the authors show that microarrays detect only a small fraction of microvesicle sequences detected by NGS, and the analysis of this greater sample reveals a definite structural pattern, namely the sequences tend to be at the 3′ end of the RNA transcripts. Since the 3′-ends of transcripts are rich in miRNA-binding sites and also contain elements conferring subcellular localization the authors suggest that exosomal RNA may act as competing RNA to regulate stability, localization and translation activity of mRNAs in recipient cells.

The paper is concisely written, the conclusions are underpinned by the data. Recently, the authors of the present paper suggested that a combination of several linear motifs can mediate targeting of secreted RNA. The present contribution represents another important step towards the structural characterization of RNA transmitted by microvesicles. The author’s suggestion that RNA delivered by exosomes plays largely regulatory roles may provoke discussions, but in my view this is what science is about.

**Authors’ response:** We appreciate the overall positive reaction of the reviewer to this work.

**Reviewer’s comment:** I find the authors have adequately addressed the issues raised previously.

## Competing interests

The authors declare that they have no competing interests.

## Authors’ contributions

AOB and IVK conceived the idea and wrote the paper. AOB performed the computational work. IVK carried out the experiments. Both authors read and approved the final manuscript.

## Supplementary Material

Additional file 1: Table S1 Gene ontologies associated with exosomal transcripts. Click here for file

Additional file 2: Figure S1 **(A)** Composition of the fragmented transcripts for probes located in the untranslated regions at ECER cutoff varying from 1 to 32. **(B)** Composition of the fragmented transcripts for probes located in the translated regions at ECER cutoff varying from 1 to 32. **(C)** Composition of the fragmented transcripts for probes located in the untranslated regions at ECER cutoff varying from 10 to 32. **(D)** Composition of the fragmented transcripts for probes located in the translated regions at ECER cutoff varying from 10 to 32. Click here for file

Additional file 3: Table S2 Comparison of the expression ratios obtained from Agilent and qPCR for selected transcripts. Click here for file

Additional file 4: Figure S2 **(A)** Distribution of individual probe expression by their relative location within the transcript/gene for weakly secreted and intracellular transcripts, ECER < 3. **(B)** Distribution of individual probe expression by their relative location within the transcript/gene for strongly secreted and intracellular transcripts, ECER ≥ 3. Click here for file

Additional file 5 Experimental part. Click here for file
