# Supplementary figures and images for: Exosomes secreted by human cells transport largely mRNA fragments that are enriched in the 3′-untranslated regions
Source: Biol Direct. 2013 Jun 7;8:12. doi: 10.1186/1745-6150-8-12 (PMC3732077; doi:10.1186/1745-6150-8-12)

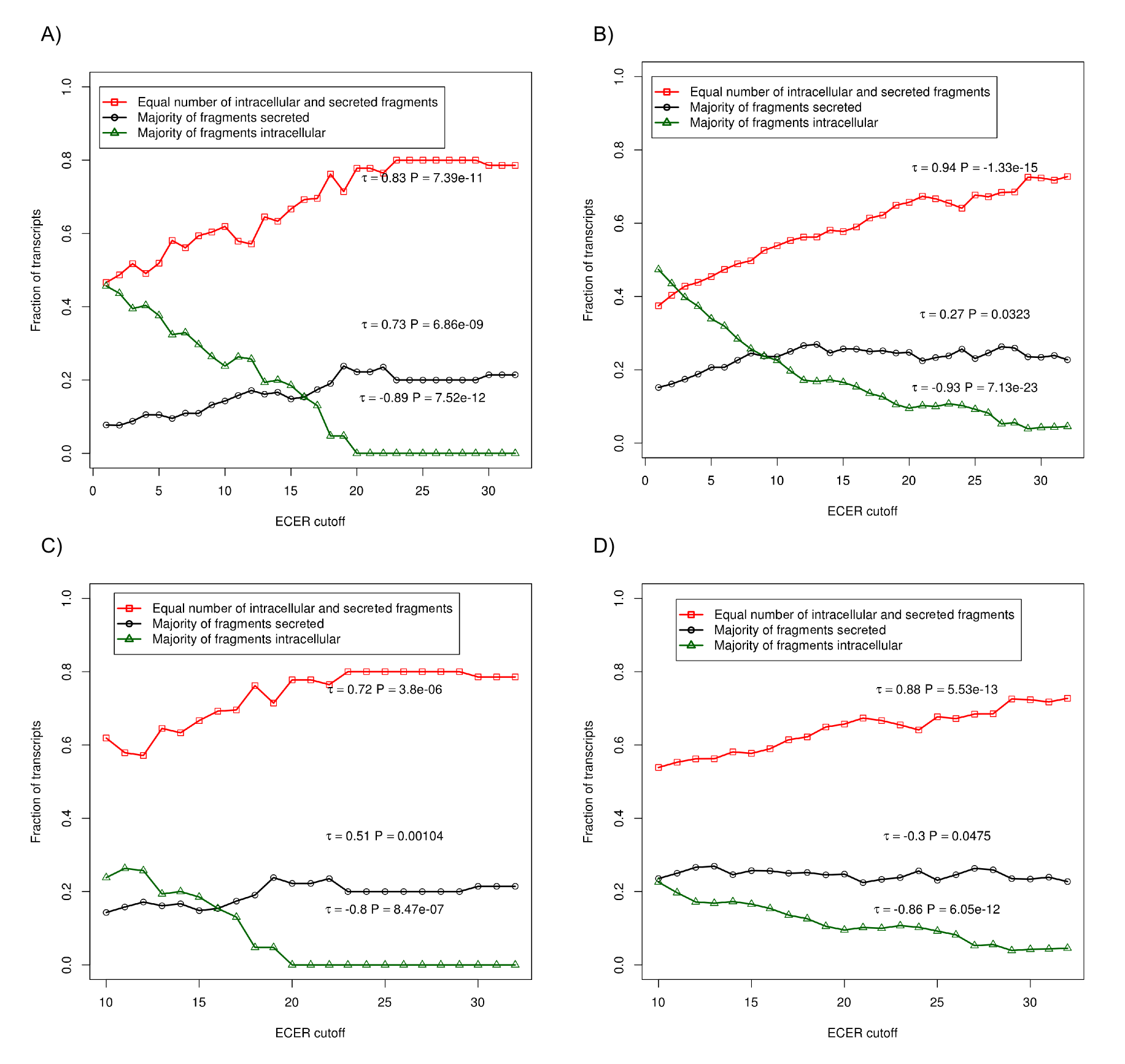

Supplement: Additional file 2: Figure S1 — (A) Composition of the fragmented transcripts for probes located in the untranslated regions at ECER cutoff varying from 1 to 32. (B) Composition of the fragmented transcripts for probes located in the translated regions at ECER cutoff varying from 1 to 32. (C) Composition of the fragmented transcripts for probes located in the untranslated regions at ECER cutoff varying from 10 to 32. (D) Composition of the fragmented transcripts for probes located in the translated regions at ECER cutoff varying from 10 to 32. [file 1745-6150-8-12-S2.png]

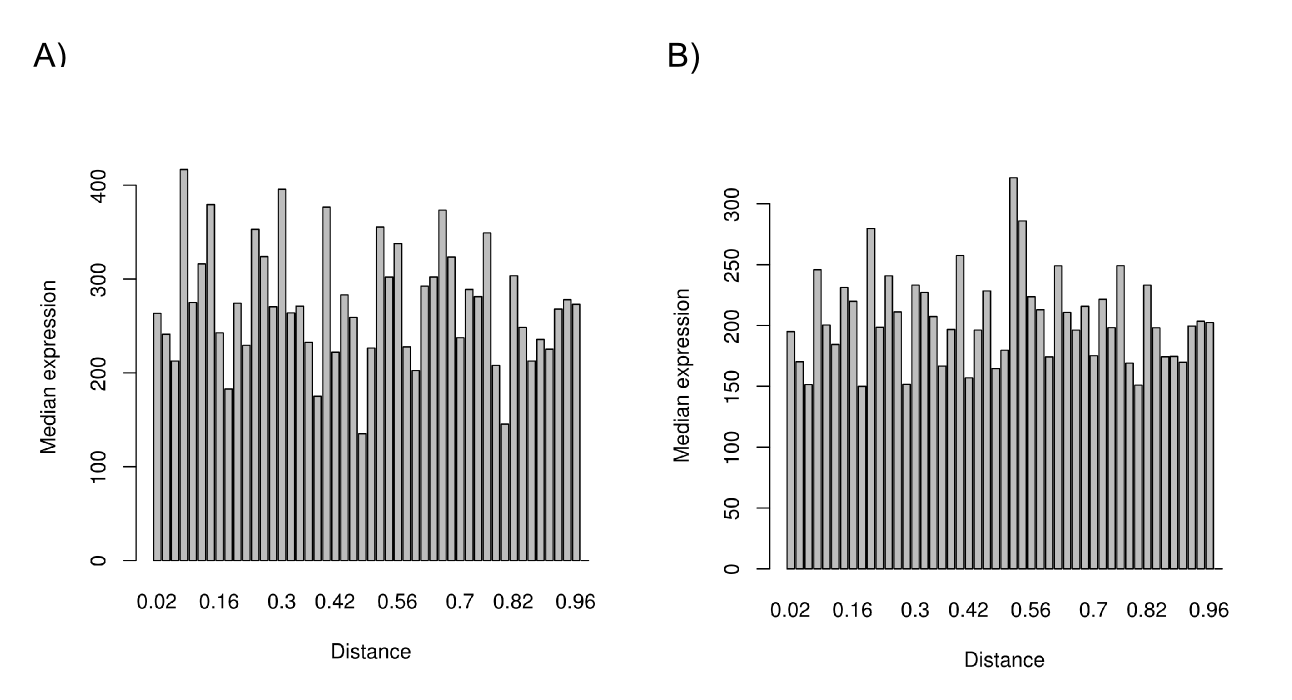

Supplement: Additional file 4: Figure S2 — (A) Distribution of individual probe expression by their relative location within the transcript/gene for weakly secreted and intracellular transcripts, ECER < 3. (B) Distribution of individual probe expression by their relative location within the transcript/gene for strongly secreted and intracellular transcripts, ECER ≥ 3. [file 1745-6150-8-12-S4.png]
