# Supplementary material for: Exosomes secreted by human cells transport largely mRNA fragments that are enriched in the 3′-untranslated regions
Source: Biol Direct. 2013 Jun 7;8:12. doi: 10.1186/1745-6150-8-12 (PMC3732077; doi:10.1186/1745-6150-8-12)
Supplement: Additional file 3: Table S2 — Comparison of the expression ratios obtained from Agilent and qPCR for selected transcripts. [file 1745-6150-8-12-S3.pdf]

Gene	Agilent					qPCR				
	Probe location	Cell	Exosome	Exosome/Cell	3' Exos/Cell 5' Exos/Cell	Amplicon location	Cell ΔC_T	Exosome ΔC_T	Exosome ΔC_T/ Cell ΔC_T	3' Exos ΔCT/Cell ΔCT 5' Exos ΔCT/Cell ΔCT
CNDP2	5'	802	587	0.732325		5'	58.89201	1.283426	0.02179287	
	3'	1396	36397	26.07235	35.60215755	3'	5.6962008	3.784231	0.664342907	30.48441594
RHO	5'	28	22	0.7857143		5'	0.9012505	0.094078	0.10438599	
	3'	276	58094	210.48551	267.8906456	3'	0.0647041	0.208772	3.226567037	30.90996253
PPFIBP1	5'	1373	107	0.0779315		5'	28.640802	1.385109	0.048361406	
	3'	1274	12868	10.100471	129.6069778	3'	16497.96	18305.63	1.109569472	22.94328397
