# Supplementary material for: Exosomes secreted by human cells transport largely mRNA fragments that are enriched in the 3′-untranslated regions
Source: Biol Direct. 2013 Jun 7;8:12. doi: 10.1186/1745-6150-8-12 (PMC3732077; doi:10.1186/1745-6150-8-12)
Supplement: Additional file 5 — Experimental part. [file 1745-6150-8-12-S5.pdf]

Additional file 5

EXPERIMENTAL PART

Cell culture. SF-295, a human glioblastoma multiforme cell line, obtained from the NCI-Frederick DCTD Tumor/Cell Line Repository, was maintained in RPMI1640-HEPES (10 mM) medium, supplemented with 10% FBS (fetal bovine serum) and GlutaMAX.

Preparing exosome-production medium. FBS-exosome-free, exosome-production medium was prepared according to Théry et al. [1]. Briefly, full medium containing 20% dialyzed FBS was centrifuged 3 hr at 120,000 x g. The supernatant (vesicle-depleted medium) was sterilized using 0.22-µm filter.

Isolation of exosome-like vesicles. SF-295 cells were grown to 90% confluency and incubated for 48 hr in in RPMI1640-HEPES (10 mM) medium, supplemented with 5% vesicle-depleted FBS. The culture supernatant was centrifuged at 300×g for 10 min to remove floating cells. The supernatant was centrifuged 17,000 x g for 25 min to remove cell debris. The vesicles were pelleted from the resulting supernatant by centrifugation at 110,000 ×g for 120 min.

RNA extraction, quantification and quality assessment. Total RNA from exosome vesicles and exosome-producing cells was extracted using Trizol (Invitrogen). The concentration of RNA in samples was measured using a NanoDrop®ND-1000 spectrophotometer. RNA size distribution was evaluated using 2100 Bioanalyzer and RNA 6000 Pico Labchip kit (Agilent Technologies; Palo Alto, CA) according to manufacturer's instructions.

cDNA synthesis and quantitative real-time PCR (RT-PCR). Two-step RT-PCR was performed using the QuantiTect Reverse Transcription Kit (QIAGEN GmbH, Hilden, Germany) according to manufacturer's recommendations. cDNA synthesis was carried out on 0.5 µg of total RNA from which genomic DNA was removed with gDNA wipeout buffer. The supplied primer mix contained a mix of oligo-dT and random primers that enabled cDNA synthesis from all regions of RNA transcripts. The firefly luciferase RNA (Promega) was reverse-transcribed identically to produce cDNA for the use as a spike-in control to evaluate the relative expression levels of various RNA regions. RT- PCR was carried out in a Rotor-Gene Q (Qiagen). Thermal cycling conditions included activation for 10 minutes at 95°C and 40 cycles of 10 seconds at 95°C, 15 seconds at 60°C, and 20 seconds at 72°C. Fluorescence data was recorded at the end of each 72°C step. A DNA melt profile was run subsequently from 72°C to 95°C with a ramp of 1°C/5 seconds. RT-PCR assays were designed for CNDP2, RHO, and PPFIBP1 genes with the amplicons of comparable length at different distances from the mRNA 3′- end (counted from the 5′ end - see table below for primer sequences). Where possible, the primers were designed in such a way that the amplified products span an exon/exon boundary. ΔC_T for a gene was calculated as a difference between C_T values for a gene of interest and that for a firefly luciferase cDNA spike-on control.

Oligonucleotide primer sequences for qPCR:

Target gene/ mRNA Accession no.	mRNA Length bp	Forward Primer (5'-3')	Reverse Primer (5'-3')	Amplicon length bp	Distance from 5'-end bp
CNDP2/	4,964	TGGAGTGCAGCAACAAAGAC	CACATCCTTGGCAAACTCCT	215	1,007
NM_001168499.1		ATCGCTCCATCTCAGTTGCT	AAGGAGCAGAGACAGGGACA	212	2,833
RHO/	2,768	TCATGGTCCTAGGTGGCTTC	GGAAGTTGCTCATGGGCTTA	186	534
NM_000539.3		GCAGGGACAGTCACAGGAAT	CCCAGGTGCTAGAGCAAAAG	231	2,196
PPFIBP1/	5,807	GCTTTTGCAGGCTTCTCAAC	ACCCGTCCTTCATCAAACTG	204	2,306
NM_001198915.1		GCACAATCTCAGCTCACTGC	CCTGGCTAACACGGTGAAAC	158	5,559
